# Exploring the Phytochemical and Physical Stability of Phycocyanin, Anthocyanins, and Betacyanin in a Cheesecake Product

**DOI:** 10.1002/mnfr.70191

**Published:** 2025-08-10

**Authors:** Cristina Selin, Anda Tanislav, Laura Stan, Bernadette‐Emőke Teleky, Călina Ciont (Nagy), Florica Ranga, Vlad Mureşan, Andruţa Mureşan, Dan Cristian Vodnar, Mihaela Mihai, Ionela Daniela Morariu, Oana Lelia Pop

**Affiliations:** ^1^ Department of Food Science University of Agricultural Sciences and Veterinary Medicine Cluj‐Napoca Romania; ^2^ Department Food Technology University of Agricultural Sciences and Veterinary Medicine Cluj‐Napoca Romania; ^3^ Molecular Nutrition and Proteomics Laboratory Institute of Life Sciences University of Agricultural Sciences and Veterinary Medicine Cluj‐Napoca Romania; ^4^ Department of Environmental and Food Chemistry University of Medicine and Pharmacy “Grigore T. Popa” Iasi Romania

**Keywords:** anthocyanins, betacyanin, cheesecake, color, phycocyanin, sweeteners

## Abstract

Color has been recognized as a paramount sensory attribute in the food industry. Although synthetic colorants have traditionally been used to ensure consistent color in food products, growing concerns about their possible toxicity and environmental impact have led to a shift toward using natural, biobased pigments. This study explored the phytochemical stability of three natural colorants (phycocyanin, anthocyanins, and betacyanin) incorporated in a cheesecake product and their interactions with various sweeteners (sucrose, fructose, sorbitol, dextrose, and xylitol). The fillings and cakes were analyzed for their phytochemical composition, color, and antioxidant properties. Phycocyanin and cyanidin‐3‐glucoside confirmed robust stability across all sweeteners. In contrast, xylitol‐sweetened cheesecake retained the highest betacyanin concentration (7.81 mg/g) and maintained it over the 5‐day shelf life (*p* > 0.05), compared to dextrose‐sweetened samples (7.07 mg/g). Dextrose and fructose significantly enhanced the stability and antioxidant properties compared to xylitol and sorbitol. These findings suggest that the choice of sweetener plays a crucial role in maintaining the stability and enhancing the health benefits of cheesecake fillings, paving the way for the development of functional foods with natural colorants.

## Graphical abstract

Phycocyanin, anthocyanins, and betacyanin were tested with various sweeteners (sucrose, fructose, dextrose, xylitol, sorbitol) to assess their impact on color retention, antioxidant activity, and sensory properties. Key parameters investigated include pigment degradation, antioxidant performance, color measurements, and consumer acceptability, offering insights into the formulation of visually appealing and nutritionally enhanced functional desserts.

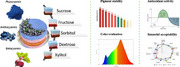

## 1 Introduction

The food industry is the foundation of the economy, profoundly impacting daily human activities and health [1]. Beyond mere sustenance, modern food production aims to promote health, prevent diet‐related diseases, and incorporate innovative technologies and ingredients [2]. This shift has driven the advancement of functional food production, emphasizing novel ingredients designed to both support overall health and mitigate risks associated with diseases such as diabetes, cancer, and cardiovascular diseases [1, 3].

Cheesecake, which is a popular dairy dessert, offers substantial nutritional value, but can also hold promise as a health‐conscious alternative when enhanced with innovative ingredients [4]. According to recent market analyses, the global industry for functional confectionery products has grown markedly, with estimates suggesting it could reach approximately $3.98 billion by 2030, reflecting a compound annual growth rate (CAGR) of around 7.1% from 2023 to 2030 [5, 6]. The incorporation of functional ingredients such as probiotics, vitamins, and antioxidants into chocolates, candies, and other sweets has broadened its product range and enhanced its appeal to consumers [5, 7, 8]. In this context, one of the pressing challenges for confectionery manufacturers is meeting the growing consumer preference for natural food coloring agents. However, they are not without challenges, as these agents must fulfill rigorous criteria, including high‐temperature stability, attractive color profiles, palatable flavors, and cost‐effectiveness [9, 10, 11].

It is considered that phenolic compounds from fruits are versatile constituents that can be used to design such novel functional products. Berries, such as strawberry *(Fragaria x ananassa*), raspberry (*Rubus idaeus* L.), blackberry (*Rubus fruticosus* L.), blueberry (*Vaccinium corymbosum* L.), red currant (*Ribes sativum* L.), and, more recently, haskap berry (*Lonicera caerulea* L.) have been reported for their substantial phenolic content [10, 12]. They are widely used as functional ingredients also due to their antioxidant and antiinflammatory activity [10, 13, 14]. Haskap berries, in particular, native to Siberia, stand out for their vibrant violet color, making them ideal for diverse food applications, from beverages to jams [12, 15]. Another color that is increasingly demanded by consumers is red. Betalains, derived from beets (*Beta vulgaris* L.), have been remarked for their striking red to violet color, making them highly desirable for various food applications. Mitrevski et al. (2023) evaluated the impact of beetroot powder incorporation on biscuits’ functional properties and shelf life [16]. Results indicate that samples baked at lower temperatures (150°C) had higher initial betacyanins values but a more statistically significant loss (57%–70%) during storage compared to samples cooked at higher temperatures (170°C) with lower beginning values and lesser loss (16%–52%) [16]. Similar findings were also suggested by [17], highlighting the influence of temperature on these pigments’ stability. In addition to fruits and vegetables, microalgae such as *Spirulina plantesis* have garnered attention for their rich protein and mineral content as well as potent pigments like C‐phycocyanin, known for its antioxidative and antiinflammatory properties [18, 19]. Despite the benefits of these natural food coloring agents, they require careful handling to maintain stability during processing and storage [20, 21].

In addition, carbohydrate structure and functionality have been reported to influence pigment stability. For instance, fructose, a highly hygroscopic reducing monosaccharide prevalent in fruit preparations, has been observed to form reversible glycosidic bonds and establish extensive hydration shells around pigment molecules. Using fructose as a sweetener in strawberry jams resulted in faster anthocyanin decomposition compared to other sweeteners like xylitol and erythritol [22]. However, fructose immersion inhibited malondialdehyde accumulation in fresh strawberries and maintained higher superoxide dismutase activity, which can extend shelf‐life by reducing oxidative stress [23]. Also, sorbitol offered a high water retention without participating in Maillard reactions. By lowering water mobility and forming hydrogen‐bond networks, sorbitol matrices restricted pigment diffusion and aggregation, preserving betacyanin chroma during thermal and refrigerated storage [24].

In light of these developments, this study aims to design and characterize a novel cheesecake recipe enriched and colored with anthocyanins from haskap berry (*L. caerulea* L.), betacyanins from red beets (*B. vulgaris* L.), and phycocyanin from *S. plantesis*. It also explores the stability of these functional pigments in conjunction with various sweeteners commonly used in confectionery. Besides their sensory roles, sweeteners are known to influence pigment stability through water activity modulation, antioxidant activity, and molecular interactions. Sucrose, fructose, and dextrose were selected to represent reducing and nonreducing sugars with varying hygroscopicities, while sorbitol and xylitol were chosen for their low glycemic index and compatibility with sugar‐free formulations.

## 2 Materials and Methods

### 2.1 Materials

All sweeteners (sucrose, fructose, dextrose, sorbitol, and xylitol) were acquired from Sigma–Aldrich (Darmstadt, Germany), and all standard compounds were 99.5% pure.

The ingredients used for cheesecake manufacturing, such as red beets (*B. vulgaris* L.), sunflower oil, powdered milk, baking powder, vanilla extract, quark cheese, beef gelatin, whipping cream (38% fat), and yogurt (2% fat), were purchased from a food store (Cluj‐Napoca, Romania). Wheat flour (0.48% ash content) and eggs were ordered from a local farm (Ileanda, Romania). The haskap berries (*L. caerulea* L. var. Vostorg) were acquired from a local family‐owned plantation (Dej, Romania). Lyophilized C‐Phycocyanin powder was provided by Merck (Germany).

For the HPLC system, acetonitrile and HPLC gradient were provided by Merck (Germany), and water was purified with a Direct‐Q UV system by Millipore (USA). The pure standard of cyanidin (purity 99% HPLC) was purchased from Sigma (USA).

The chemicals for the biological characterization, DPPH (1,1‐diphenyl‐2‐picrylhydrazyl), ABTS radical scavenging capacity (2,2′‐azinobis‐(3‐ethylbenzothiazoline‐6‐sulfonic acid)), and Trolox, were also procured from Sigma–Aldrich (Germany). Culture media components and other reagents were from VWR International (Radnor, Pennsylvania, PA, USA). All the materials and chemicals used in the experiment were of analytical grade.

### 2.2 Cheesecake Formulation

The preparation of the cheesecakes involved three main steps: crafting the cake, formulating the filling, and assembling the final product. In each formulation, the same sweetener (sucrose, fructose, sorbitol, dextrose, and xylitol) was used both in the cake base and the filling. The recipes for the five sweetener formulations (sucrose, fructose, sorbitol, dextrose, and xylitol) are presented in Supporting Information File S1 (Table S1) and refer to 100 g of each cheesecake product. The sucrose‐sweetened cheesecake was used as the control to provide a standard reference point for the most common sugar typically used in confectionery.

#### 2.2.1 Beetroot By‐Product Preparation

Beetroots were processed for juice extraction, and the resulting by‐product was subsequently utilized for cake manufacturing. The by‐product underwent additional pressing and blending to remove residual juice from the pulp completely. The quantity of by‐products added to the cake was determined based on the organoleptic properties observed during preliminary testing. The freshly obtained by‐product was used for future incorporation in cake formulation.

#### 2.2.2 Freeze‐Drying Process of Berries

Fresh haskap berries (*L. caerulea* L. var. Vostorg) (200 g) were first washed and then frozen at target temperatures (−80°C), according to a previous study by [25]. Further, the frozen samples were dried in a lyophilizer (Telstar LyoQuest, Terrassa, Azbil Group, Spain). The ice condenser was cooled to −55°C, and the pressure was set to 0.001 mBar. After 72 h of freeze‐drying, the dried fruits underwent processing through grinding and sieving to achieve a finely powdered form. The powder was kept in an airtight, lightless container for further use in the cheesecake formulation.

#### 2.2.3 Cheesecake Manufacturing

For the cake, egg albumens were whipped with each sweetener using a Duty KitchenAid mixer, and white flour and baking powder were mixed separately. Subsequently, egg yolks and sunflower oil were incorporated into the whipped egg albumens, followed by the addition of beetroot pulp and the flour mixture. The resulting composition was baked at 200°C for 20 min in an electric professional oven (Venix T04MP) and cooled at room temperature.

For the filling preparation, whipping cream was aerated in a Duty KitchenAid mixer, while gelatin was hydrated and combined with yogurt on the Bartscher Professional Electric Stove to reach 35°C. Quark cheese, sweetener, yogurt–gelatin mixture, and vanilla extract were blended until homogeneous, after which whipped cream was folded. Finally, the cream was split into two portions: berries powder was incorporated for the pink filling, and phycocyanin powder was added for the blue filling.

The assembly involved placing the cake into a 19 cm stainless steel cake mold, then layering the berry filling (pink), followed by the phycocyanin filling (blue). The cheesecake was then refrigerated at 4°C for 24 h and afterward for further characterization and analysis. The samples were codified as follows: SuC for sucrose‐sweetened cheesecake, FC for fructose‐sweetened cheesecake, SoC for sorbitol‐sweetened cheesecake, DC for dextrose‐sweetened cheesecake, and XC for xylitol‐sweetened cheesecake. The fillings were further identified by color and type: SuC_P for sucrose‐sweetened pink filling, FC_P for fructose‐sweetened pink filling, SoC_P for sorbitol‐sweetened pink filling, DC_P for dextrose‐sweetened pink filling, XC_P for xylitol‐sweetened pink filling, SuC_B for sucrose‐sweetened blue filling, FC_B for fructose‐sweetened blue filling, SoC_B for sorbitol‐sweetened blue filling, DC_B for dextrose‐sweetened blue filling, XC_B for xylitol‐sweetened blue filling, SuC_C for sucrose‐sweetened cake, FC_C for fructose‐sweetened cake, SoC_C for sorbitol‐sweetened cake, DC_C for dextrose‐sweetened cake, and XC_C for xylitol‐sweetened cake. The nutritional, microbiological, and sensory analyses were conducted on the overall cheesecakes, whereas the phytochemical stability, pH, colorimetric, antioxidant, and rheological analyses were performed separately for each filling.

### 2.3 Nutritional Composition

The nutritional properties of the cheesecakes were obtained using the AACC (2000) methods [26]. Moisture (AACC 44‐15.02, 2000), fat (AACC 30‐25.01, 2000), and ash (AACC 08‐01.01, 2000) contents were estimated by employing the standard analysis method. The protein content was detected according to the Kjeldahl method (AACC 46‐11.02, 2000), a nitrogen‐to‐protein conversion factor of 5.7.

The total carbohydrate (%) content was calculated using Equation (1) [27].

(1) $$\begin{array}{cc} & \mathrm{Total}\;\mathrm{carbohydrate}\;\left(\%\right)\\ & \;=100-\left[\mathrm{moisture}\left(\%\right)+\mathrm{ash}\left(\%\right)+\mathrm{proteins}\left(\%\right)+\mathrm{lipids}\left(\%\right)\right] \end{array}$$

The total calories were calculated using Atwater's conversion factor to establish the energy content derived from fat, protein, and carbohydrates. The caloric content for sucrose, fructose, and dextrose‐sweetened cheesecake was calculated using Equation (2).

(2) $$\begin{array}{cc} & \mathrm{Energy}\;\mathrm{value}\;\left(\mathrm{kcal}/100\;g\right)\\ & \;=\left(\mathrm{Carbohydrates}\;\times \;4.0\right)+\left(\mathrm{Protein}\;\times \;4.0\right)+\left(\mathrm{Fat}\;\times \;9.0\right) \end{array}$$

In the case of xylitol and sorbitol‐sweetened cheesecake, the energy value was estimated using 2.4 kcal/g [28] for the portion of carbohydrates following Equation (3).

(3) $$\begin{array}{cc} & \mathrm{Energy}\;\mathrm{value}\;\left(\mathrm{kcal}/100\;g\right)\\ & \;=\left(\mathrm{Carbohydrates}\;\times \;2.4\right)+\left(\mathrm{Protein}\;\times \;4.0\right)+\left(\mathrm{Fat}\;\times \;9.0\right) \end{array}$$

### 2.4 Microbiological Analysis

For the microbiological analysis, 3 g of each of the five cheesecakes were mixed with 27 mL of sterile saline (0.85% NaCl) and blended for 2 min using a Stomacher Lab Blender 80 (London, UK). Samples included the entire cheesecake product for a comprehensive assessment. *Escherichia coli* was identified on TBX Agar at 44°C for 24 h, *Staphylococcus aureus* on BPA with Egg Yolk Tellurite Emulsion at 37°C for 48 h, *Enterobacteriaceae* on VRBG Agar at 37°C for 24 h, yeasts and molds on DRBC Agar at 25°C for 5 days, and coliform bacteria on VRBL at 37°C for 24 h. Analyses were performed in triplicate for each independent cheesecake sample, separately for each sweetener group, at three storage points (4°C): Day 2 (market readiness), Day 5 (expiration date), and Day 7 (extended safety period). Results were reported as log CFU/g.

### 2.5 Phytochemical Stability of the Pigments in Cheesecakes

#### 2.5.1 Extraction of Pigments

##### 2.5.1.1 Phycocyanin

For phycocyanin extraction, 5 g of blue filling from each cheesecake type was dissolved in 10 mL of 0.1 M phosphate buffer at pH 7.0 [19]. The mixture was ultrasonicated at 37 kHz for 30 min in an Elma Schmidbauer (Singen, Germany) ultrasonic bath, then centrifuged at 12 298 × *g* for 10 min. The supernatant containing the extracted phycocyanins was filtered through a 0.45 µm Millipore filter (Sigma–Aldrich) and prepared for further analysis.

##### 2.5.1.2 Anthocyanins

A 5 g sample of pink filling from various cheesecakes was homogenized with 10 mL of 95% methanol containing 0.01% HCl [29]. The mixture was ultrasonicated for 15 min, then centrifuged at 12 298 × *g* for 10 min. The supernatant was evaporated at 35°C under low pressure using a Buchi Rotavapor R‐124 (Flawil, Switzerland), then filtered through a 0.45 µm Millipore filter for future analyses. All procedures were performed in low light and under controlled conditions to prevent anthocyanin degradation.

##### 2.5.1.3 Betacyanins

Extraction and analysis of betacyanins from the cakes of each type of cheesecake were carried out as described previously by [13]. Briefly, 5 g of the cake was homogenized with 10 mL of Milli‐Q water using an Ultra‐Turrax IKA T18 151 Basic (Wilmington, USA) at 12 298 × *g* for 15 min in an ice bath to avoid thermal degradation. Following homogenization, samples underwent sonication for 10 min to enhance pigment release from the dense cake matrix and were then centrifuged at 12 298 × *g* at 4°C for 10 min. The clear supernatant was collected and filtered through a 0.45 µm Millipore membrane. Betacyanin content was subsequently quantified spectrophotometrically as described in Section 2.5.2.

#### 2.5.2 Quantitative Analysis

##### 2.5.2.1 Determination of Phycocyanin Content

The phycocyanin extracts were examined spectrophotometrically using a UV–Vis Spectrophotometer (Jasco V‐630, International Co. Ltd, Japan) at 615 and 682 nm. The concentration of the phycocyanin pigment was determined according to Equations (4) as indicated as follows:

(4) $$\mathrm{Phycocyanin}\;\mathrm{concentration}\;\left(\mathrm{mg}/g\right)=\frac{A_{615}-0.474\times A_{652}\;}{5.34}$$

where *A*_615_ is the absorbance at 615 nm; *A*_652_ is the absorbance at 615 nm.

##### 2.5.2.2 Determination of Total Anthocyanin Content

The differential pH technique was used to quantify the total anthocyanin content expressed in cyanidin‐3‐glucoside equivalents [29]. The dilution of each extract was performed at a ratio of 1:5 with sodium acetate buffer (0.4 M) at pH 1.0 and 4.5. The incubation of the diluted extracts was carried out in darkness for 15 min. The extracts were then subjected to spectrophotometric analysis using a UV–Vis Spectrophotometer to measure their absorbance at 535 nm wavelengths. The total anthocyanin values were determined by using the following equation:

(5) $$\mathrm{Anthocyanins}\;=\frac{A\;\times \;\mathrm{MW}\;\times \;1000}{\varepsilon x}$$

The results were expressed as mg cyanidin‐3‐glucoside equivalent per 100‐g weight sample using its molar absorptivity value of 34 300 in HCl (*ε*) and 484.8 for its molecular weight (MW). *A* is the absorbance.

##### 2.5.2.3 Determination of Betacyanins Content

The betacyanin content was measured using UV–Vis spectrophotometry by recording the absorbance of the supernatant at 538 nm [30]. The concentration (mg/100‐g weight sample) was calculated according to Equation (6):

(6) $$\mathrm{Betacyanins}\;=\frac{A\;\times \mathrm{MW}\;\times \;1000}{\varepsilon \;\times \;L}$$

where *A* is the absorbance at 538 nm; MW the 550.473 g/mL; *ε* the 65 000 L/mol; and *L* (path length) is the 1.0 cm.

#### 2.5.3 Anthocyanins Separation and Identification Using HPLC–DAD–ESI–MS

The analyses were conducted using an HP‐1200 liquid chromatograph equipped with a quaternary pump, autosampler, DAD detector, and MS‐6110 single quadrupole API‐electrospray detector (Agilent‐Techonologies, USA). The positive ionization mode was applied to detect the phenolic compounds; different fragments in the 50–100 V range were used. The column was a Kinetex XB‐C18 (5 µm; 4.5 × 150 mm i.d.) from Phenomenex, USA. The mobile phase was (A) water acidified by formic acid 0.1 % and (B) acetonitrile acidified by formic acid 0.1%. The following multistep linear gradient was applied: start with 5% B for 2 min; from 5% to 90% of B in 20 min, hold for 4 min at 90% B, then 6 min to arrive at 5% B. Total analysis time was 30 min, flow rate 0.5 mL/min, and oven temperature 25 ± 0.5°C. Mass spectrometric detection of positively charged ions was performed using the Scan mode. The applied experimental conditions were: gas temperature 3500°C, nitrogen flow 7 L/min, nebulizer pressure 35 psi, capillary voltage 3000 V, fragment 100 V, and *m*/*z* 120–1500. Chromatograms were recorded at wavelength *λ* = 520 nm, and data acquisition was complete with the Agilent ChemStation software, Rev B.02.01‐SR2 [260] version.

#### 2.5.4 pH Level

A WTW inoLab 7110 laboratory pH meter equipped with a conical penetration probe designed for measuring semi‐solid and solid food products was used to measure the pH of the distinct cheesecake fillings (blue, pink, and cake sections). At each sampling time, three pieces from the cheesecakes were measured in six locations. The sampling locations were arranged in a triangle pattern at an equal distance apart on the top and bottom of the cheesecakes.

#### 2.5.5 Colorimetric Analysis

The colorimetric determination of the cheesecakes was performed by measuring the parameters L*, a*, and b* using a portable colorimeter NR200 (3NH, Shenzhen, China) that performs automatic calibration. The parameter L* measures the lightness of the sample, with values from 0 to 100, a* the values from green (−*a*) to red (+*a*) (−128…+128), and b* from blue (−*b*) to yellow (+b) (−128…+128). The analyses were made for the cheesecake fillings (blue and pink) and the cake crust (section and external surface). The colorimetric determination was performed in six repetitions for each sample during the 5 days of storage.

### 2.6 Antioxidant Activity Assays

#### 2.6.1 DPPH Activity

The antioxidant activity and free radical scavenging capacity of the extracts were evaluated using the DPPH assay, following a previously described method [14]. Briefly, 250 µL of DPPH solution was combined with 35 µL of the cheesecake extracts and incubated in the dark at room temperature for 30 min. The absorbance was then measured at 515 nm using a microplate reader (BioTek Instruments, Winooski, VT, USA). Antioxidant effectiveness was calculated using the following Equation (7):

(7) $$\mathrm{DPPH}\;\mathrm{scavenging}\;\mathrm{activity}\;\left(\%\right)=\frac{A_{b}-A_{s}}{A_{b}}\;\times \;100,$$

where *A*_s_ represents the absorbance of the sample and *A*_b_ represents the blank absorbance.

#### 2.6.2 ABTS Radical Cation Decolorization Assay (*ABTS* ^+^)

The antioxidant activity of the above extracts prepared was determined according to the procedure described by Arnao et al. [31]. The blue‐green ABTS^+^ solution was freshly prepared by mixing a 7 mM aqueous solution of ABTS^+^ and 2.45 mM potassium persulfate. ABTS^+^ working solution was obtained by diluting the stock solution with ethanol, having an absorbance of 0.700 ± 0.02 AU at 734 nm. After obtaining the working solution, 20 µL of samples at different concentrations of cheesecake by‐product extracts were added to 170 µL ABTS^+^ solutions, incubated for 6 min at room temperature in the dark, and the absorbance was measured using a microplate reader. The antioxidant activity was calculated as presented in Equation (8):

(8) $$\mathrm{ABTS}\;\mathrm{radical}\;\mathrm{scavenging}\;\mathrm{activity}\;\left(\%\right)=\frac{A_{b}-A_{s}}{A_{b}}\times 100$$

where *A*_b_ is the absorbance of the blank, and *A*_s_ is the samples absorbance.

### 2.7 Rheological Measurements

An Anton Paar MCR 72 rheometer (Anton Paar, Graz, Austria), equipped with a Peltier plate‐plate system (P‐PTD 200/Air), was utilized to measure the viscosity of the distinct cheesecake fillings of the (blue, pink, and cake section). Samples were placed between the two plates, with the lower plate maintained at a temperature of 4°C and a gap of 1 mm, while the upper plate featured a smooth parallel plate geometry with a 50 mm diameter [32]. Any excess sample was removed to ensure thermal equilibrium before measurement, and the samples were allowed to rest for 10 min. Viscosity measurements were conducted twice for each sample with a linearly increasing shear rate ranging from 5 to 300 s^−1^.

### 2.8 Sensory Analysis

A hedonic test (ISO 11136:2014) of the cheesecake samples (SuC, FC, SoC, DC, and XC) was conducted by a sensory panel comprising 14 voluntary evaluators (64% females and 36% men) aged between 24 and 46 years old (mean value of 28.43 ± 5.93), at the University of Agricultural Sciences and Veterinary Medicine of Cluj‐Napoca, Romania [33]. Sensory evaluation followed the laboratory ethical standards, and each evaluator signed a written informed consent [27]. All participants were fully informed about the product type and ingredients, the aim of the sensory test, and the voluntary nature of their participation. Only those who had no allergies or intolerances to any of the ingredients and who agreed to sign the consent form were included in the test. We confirm that ethical permission was obtained for this study. The appropriate protocols to protect the rights and privacy of all participants were strictly followed throughout the research process. The samples were coded uniquely and anonymously, using three distinct digit codes, and presented in random order. Plain mineral water was provided to clean the palate between samples. The samples were provided cold to the evaluators (∼±4°C). During the sensory analysis, a break was provided between samples to avoid sensory fatigue. Samples were evaluated for 11 sensory characteristics – exterior surface, odor, color, texture after cutting, taste, sweetness, acidity, aroma, bitterness, off taste, and after taste using a nine‐point hedonic scale where: (1) dislike extremely, (2) dislike very much, (3) dislike moderately, (4) dislike slightly, (5) neither like nor dislike, (6) like slightly, (7) like moderately, (8) like very much, and (9) like extremely. Data were interpreted by calculating the mean value and standard deviation (SD) for each sensory attribute.

### 2.9 Statistical Analysis

All measurements and analyses were made on three prepared samples, and the results are presented as means ± SD. One‐way analysis of variance (ANOVA) and Tukey's comparison test via Minitab statistical software (version 16.1.0; LEAD Technologies, Inc., Charlotte, NC, USA) were applied to analyze the differences among samples with significance levels of *p* < 0.05. Statistical significance was assumed at the 95% confidence level for differences in mean values.

## 3 Results and Discussions

The interaction between pigments and sugars was analyzed to assess the influence of sugar on pigment stability in food products. Spectrophotometric analysis provided real‐time data on how pigments responded to various sugars over time. Additionally, nutritional, sensory, microbiological, and rheological evaluations were conducted to determine the overall impact of sugars.

### 3.1 Nutritional Composition

The nutritional composition of the cheesecakes with different sweeteners is presented in Table 1. In essence, the choice of sweeteners in cheesecake recipes can slightly impact the nutritional content. The selection of sweeteners resulted in minor variations in the overall nutritional profile. However, the incorporation of different sweeteners had a significant effect on the moisture content of the final products. These differences in moisture levels are likely due to the distinct physicochemical properties of each sweetener. Sorbitol and xylitol have been associated with highly hygroscopic properties, and they are commonly used in sugar‐free products (gums and candies) to maintain moisture and prevent drying out of the products [34]. The protein content remained significantly consistent across all samples, with a slightly lower protein concentration observed in the XC. In terms of fat content, the values were closer among all the samples. These observations align with other studies that have explored the impact of different sweeteners on protein and fat levels in food products, highlighting their minimal effect on these nutrients [2, 35]. Since all the sweeteners are pure carbohydrates or sugar alcohols [34], the influence on ash content varied insignificantly among the samples. Carbohydrate content was highest in the SoC and lowest in the DC. This observation aligned with research on using low MW carbohydrates such as glucose, fructose, and dextrose as sweeteners in pharmaceutical products [36].

**TABLE 1:** Nutritional composition of the cheesecakes with different sweeteners.

Nutritional parameters	Cheesecake samples				
	SuC	FC	SoC	DC	XC
Moisture (%)	49.95^C^ ± 0.06	52.27^B^ ± 0.03	53.52^A^ ± 0.03	48.36^D^ ± 0.06	53.52^A^ ± 0.01
Protein (%)	5.83^A^ ± 0.10	5.80^A^ ± 0.20	5.78^A^ ± 0.10	5.79^A^ ± 0.20	5.26^B^ ± 0.10
Fat (%)	12.44^B^ ± 0.12	12.01^D^ ± 0.10	12.23^C^ ± 0.11	12.44^B^ ± 0.16	12.89^A^ ± 0.10
Ash (%)	1.46^AB^ ± 0.20	1.89^A^ ± 0.05	1.17^B^ ± 0.09	1.26^B^ ± 0.05	1.55^AB^ ± 0.08
Total carbohydrate (%)	30.22^C^ ± 0.49	32.57^B^ ± 0.06	34.34^A^ ± 0.14	28.87^C^ ± 0.11	33.82^B^ ± 0.09
Energy value (kcal/100 g)	256.16^A^ ± 0.20	261.57^A^ ± 0.02	215.60^C^ ± 0.44	250.60^B^ ± 0.57	218.21^C^ ± 0.48

*Note*: All data are the mean ± SD of three independent determinations. Mean followed by different letters in the same row differs significantly (*p* < 0.05).

Abbreviations: DC, dextrose sweetened cheesecake; FC, fructose‐sweetened cheesecake; SoC, sorbitol sweetened cheesecake; SuC, sucrose sweetened cheesecake; XC, xylitol sweetened cheesecake.

### 3.2 Microbiological Analysis

*Enterobacteriaceae*, *E. coli*, *S. aureus*, and coliform bacteria analyses were performed to determine the microbiological proprieties of the five cheesecake samples during storage for 7 days at 4°C. The findings showed that these counts were below the detection limit. Yeasts‐mold (Y&M) results are presented in Supporting Information File S1 (Table S2). The Y&M increased during shelf life, but the changes were not significant (*p* < 0.05). The results confirm the capacity of some sweeteners (sorbitol and xylitol) to offer some antifungal benefits due to their hyperosmotic properties, making them advantageous in reducing microbial activity in food products [37, 38].

### 3.3 Phytochemical Stability of the Pigments in Cheesecakes

**FIGURE 1:**
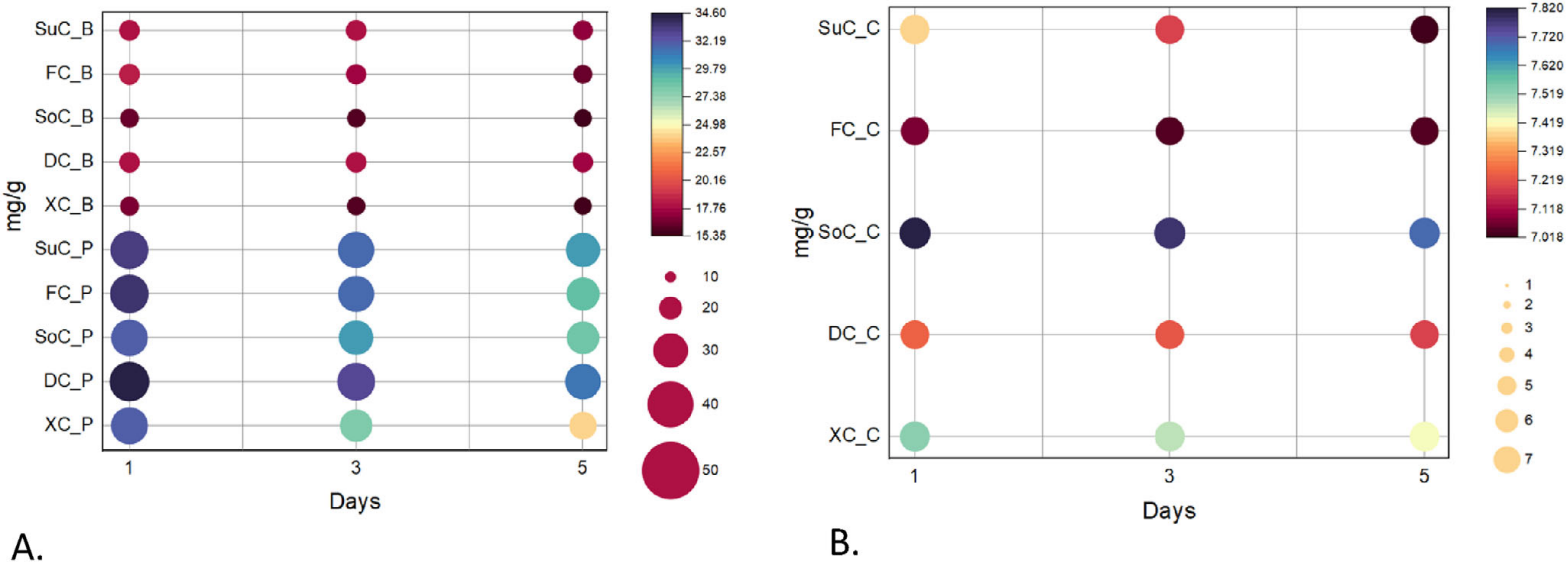
Bubble matrix representation of pigment stability of each filling cheesecake (A) and (B) of every cheesecake cake. The data are displayed on a color scale with red color for the lowest level, lighter red for the higher level toward the average level in white, and green for above average toward the highest level in dark blue. DC_B, dextrose‐sweetened cheesecake filling – blue; DC_C, dextrose‐sweetened cheesecake cake; DC_P, dextrose‐sweetened cheesecake filling – pink; FC_B, fructose‐sweetened cheesecake filling – blue; FC_C, fructose‐sweetened cheesecake cake; FC_P, fructose‐sweetened cheesecake filling – pink; SoC_B, sorbitol‐sweetened cheesecake filling – blue; SoC_C, sorbitol‐sweetened cheesecake cake; SoC_P, sorbitol‐sweetened cheesecake filling – pink; SuC_B, sucrose‐sweetened cheesecake filling – blue; SuC_C, sucrose‐sweetened cheesecake cake; SuC_P, sucrose‐sweetened cheesecake filling – pink; XC_B, xylitol‐sweetened cheesecake filling – blue; XC_C, xylitol‐sweetened cheesecake cake; XC_P, xylitol‐sweetened cheesecake filling – pink.

The stability of each pigment (phycocyanin, anthocyanins, and betacyanin) was evaluated during cheesecake shelf life (Figure 1). Phycocyanin and anthocyanins fillings show varying stability levels between the different sweeteners used. For the phycocyanin filing, no significant differences were observed over the 5‐day monitoring period across different sweeteners (*p* > 0.05). This aligns with previous studies that have demonstrated the robustness of phycocyanin under neutral conditions [9, 11, 39]. Phycocyanin was directly related to the color quality of food and served as a valuable functional ingredient [18]. For instance, Yin et al.’s results indicate that phycocyanin undergoes denaturation and degradation at a pH of 4, making it unsuitable for acidic food products [11]. On the other hand, previous studies have reported that high concentrations of monosaccharides and disaccharides can improve phycocyanin stability [9, 39]. These sugars can prevent the oxidative degradation of this pigment by binding with protein via an N‐linked glycosidic bond, scavenging free radicals, generating a protective viscous layer, and chelating metal ions that catalyze oxidative reactions [9, 18, 40]. For this reason, Hadiyanto et al. investigated the effects of different types of sugars as phycocyanin stabilizers. The results indicate that sucrose has a complex structure and has difficulty bonding with protein due to the O‐linked glycosidic bond between its glucose and fructose structures, explaining why it is known as a nonreducing sugar. Conversely, fructose could easily bond with protein in phycocyanin by a glycosidic bond [18]. Thus, the stability of phycocyanin can be affected by the pH and sugar–protein interaction via the glycosidic bond, which could polymerize phycocyanin and prevent degradation reactions [11, 18].

**FIGURE 2:**
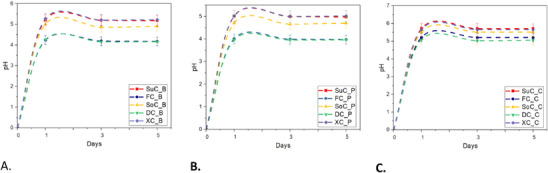
pH evaluation of each filling cheesecake (A, B) and of every cheesecake cake (C). The results are expressed as means ± standard deviation. DC_B, dextrose‐sweetened cheesecake filling – blue; DC_C, dextrose‐sweetened cheesecake cake; DC_P, dextrose‐sweetened cheesecake filling – pink; FC_B, fructose‐sweetened cheesecake filling – blue; FC_C, fructose‐sweetened cheesecake cake; FC_P, fructose‐sweetened cheesecake filling – pink; SoC_B, sorbitol‐sweetened cheesecake filling – blue; SoC_C, sorbitol‐sweetened cheesecake cake; SoC_P, sorbitol‐sweetened cheesecake filling – pink; SuC_B, sucrose‐sweetened cheesecake filling – blue; SuC_C, sucrose‐sweetened cheesecake cake; SuC_P, sucrose‐sweetened cheesecake filling – pink; XC_B, xylitol‐sweetened cheesecake filling – blue; XC_C, xylitol‐sweetened cheesecake cake; XC_P, xylitol‐sweetened cheesecake filling – pink.

In contrast, the anthocyanin filling exhibited a more pronounced degradation rate (*p* < 0.05), with the highest degradation occurring in the XC_P and the most protection observed in the DC_P. This can be attributed to the higher pH levels recorded in the XC_P filling (Figure 2), which accelerate cyanidin‐3‐glucoside degradation, resulting in the highest denaturation rate (25.63%). HPLC chromatogram analysis (Supporting Information File S1 (Figure S1) confirmed that cyanidin‐3‐glucoside was the only anthocyanin detected in all the pink extracts. The acidic pH range of the DC_P and FC_P resulted in greater stability of this pigment. This observation is supported by other studies [10, 41], which indicate increased pigment stability in acidic conditions. Moreover, the properties of sugars can influence the degradation of cyanidin‐3‐glucoside [42, 43]. The effect of sugars on anthocyanin stability has been shown to depend on factors such as water content and availability in the medium, along with the type and concentration of the sugar [43]. The rise in water availability increased the breakdown of anthocyanins owing to the assault of water molecules [43, 44]. Conversely, the increase in sugar concentration caused a reduction in the breakdown of anthocyanins [9]. The findings of Ertan et al. suggest that the stabilities of cyanidin‐3‐glucoside and color density in strawberry nectars sweetened with honey (fructose 38.38%) were higher than those sweetened with sucrose and maltose syrup [7]. Also, another study indicates that dextrose may prevent the hydrolysis of this pigment by stabilizing the glycosidic bond, which helps maintain the molecule integrity under oxidative stress [42]. This hydration shell can shield the glycosidic bond from hydrolytic attacks by reducing the direct interaction of water molecules with the bond [43, 44]. In summary, the stability of cyanidin‐3‐glucoside in anthocyanin fillings was influenced by pH and sugar type, with acidic conditions and certain sugars (fructose and dextrose) enhancing stability and preventing degradation.

The thermal stability of betacyanins in the cakes was markedly influenced by the type of sweetener incorporated. Betacyanin concentrations ranged from 7.81 to 7.07 mg/g, with the highest level observed in the XC_C formulation. The different interaction between each sweetener and the food matrix affects the thermal degradation rate of betacyanins. Sugar alcohols such as sorbitol and xylitol have demonstrated a protective effect against thermal degradation, attributed to their strong water‐binding capacity and their nonparticipation in Maillard reactions [45, 46]. First, the polyol structure of xylitol enables strong hydrogen‐bonding with water, reducing free water activity and thus limiting pigment hydrolysis and oxidative cleavage [47]. Second, as a nonreducing sweetener, xylitol has been reported not to participate in Maillard reactions that can generate reactive carbonyl species, minimizing pigment–carbonyl interactions that lead to degradation [48]. Third, the highly viscous microenvironment created by xylitol physically restricts pigment diffusion and shields betacyanin molecules from oxygen and light exposure [47, 48]. The study on reduced‐calorie sponge cakes found that using maltitol and oligofructose as alternative sweeteners helped maintain the color and appearance of the cakes compared to regular sugar [46]. In the same way, Kim et al. evaluated the thermal stability of sugar alcohols in a rice chiffon cake. The results showed that sugar replacement improved batter stability by 30% over sugar cake and its specific volume, allowing more air retention than other polyols and developing equal‐cell networks [8].

However, pigment stability was not significantly influenced throughout the 5‐day shelf life period of the cheesecakes (*p* > 0.05). Betacyanin degradation was primarily associated with the baking step during cake base preparation, consistent with the known thermal instability of betalains, which are prone to dehydrogenation, decarboxylation, and isomerization reactions at temperatures exceeding 50–60°C [49]. The thermal degradation was also observed visually through significantly fading the initial vibrant reddish‐purple hue in the baked cake matrix. Thermal degradation was also evident through the noticeable fading of the initial vivid reddish‐purple coloration in the baked cake matrix. Additionally, the pH values of the cakes remained stable across all observation periods. These findings suggest that the choice of sweetener plays a critical role in maintaining the color integrity and overall stability of betacyanins during thermal processing and shelf life.

### 3.4 Colorimetric Analysis

Color is a critical attribute in the development of novel food products, as it greatly influences consumer purchasing behavior. To assess color stability, the samples were analyzed over a 5‐day storage period, beginning 1‐day postproduction. Variations in the type of sweeteners incorporated into the fillings and crusts affected the colorimetric parameters (L*, a*, b*). The corresponding color values for the cheesecake fillings are summarized in Table 2.

**TABLE 2:** Color characteristics for cheesecake fillings with different sweeteners.

		SuC_P	FC_P	SoC_P	DC_P	XC_P	SuC_B	FC_B	SoC_B	DC_B	XC_B
Day 1	L*	56.53^A^ ± 0.62	57.21^A^ ± 0.50	56.39^A^ ± 1.48	49.26^B^ ± 1.14	58.50^A^ ± 11.95	60.42^A^ ± 0.90	58.94^B^ ± 0.56	59.07^AB^ ± 0.45	56.97^B^ ± 0.63	59.08^AB^ ± 1.16
	a*	15.29^A^ ± 0.39	14.27^A^ ± 0.38	11.74^B^ ± 0.23	14.98^B^ ± 0.31	11.95^B^ ± 0.15	0.18^A^ ± 1.19	−1.27^B^ ± 0.27	−2.01^A^ ± 0.09	−1.94^B^ ± 0.62	−2.26^B^ ± 0.23
	b*	11.01^A^ ± 0.30	10.91^A^ ± 0.17	10.36^A^ ± 0.45	7.87^B^ ± 0.21	10.94^A^ ± 0.15	3.94^A^ ± 0.58	1.13^A^ ± 0.19	1.61^A^ ± 0.13	−0.67^A^ ± 0.22	0.83^A^ ± 0.19
Day 2	L*	52.98^B^ ± 0.77	55.15^B^ ± 0.31	54.47^B^ ± 0.79	47.69^C^ ± 0.63	57.31^AB^ ± 0.99	58.34^B^ ± 1.40	57.80^BC^ ± 0.68	57.95^BC^ ± 1.44	56.45^B^ ± 0.54	59.75^A^ ± 0.84
	a*	12.10^B^ ± 0.45	12.17^B^ ± 0.15	11.78^B^ ± 0.24	15.25^B^ ± 0.25	11.64^B^ ± 0.25	−2.31^C^ ± 0.21	−2.71^C^ ± 0.07	−2.91^B^ ± 0.12	−3.19^C^ ± 0.15	−2.72^B^ ± 0.15
	b*	8.99^B^ ± 0.21	9.38^B^ ± 0.08	9.30^C^ ± 0.15	6.91^C^ ± 0.15	9.94^C^ ± 0.11	2.49^BC^ ± 0.25	−0.17^B^ ± 0.12	0.51^BC^ ± 0.09	−1.43^B^ ± 0.25	0.79^A^ ± 0.10
Day 3	L*	55.62^A^ ± 0.61	57.36^A^ ± 0.50	56.07^A^ ± 0.25	51.34^A^ ± 0.28	58.68^A^ ± 0.93	60.79^A^ ± 0.45	61.93^A^ ± 1.22	60.07^A^ ± 0.69	59.03^A^ ± 0.62	60.42^A^ ± 0.57
	a*	14.82^A^ ± 1.12	14.49^A^ ± 0.80	12.43^A^ ± 0.32	17.80^A^ ± 0.51	12.70^A^ ± 0.35	−1.13^B^ ± 0.21	1.58^A^ ± 0.90	−2.00^A^ ± 0.35	−0.54^A^ ± 0.28	−0.95^A^ ± 0.57
	b*	10.58^A^ ± 0.53	10.73^A^ ± 0.50	9.91^AB^ ± 0.14	8.76^A^ ± 0.28	10.65^B^ ± 0.29	2.88^B^ ± 0.13	1.05^A^ ± 0.55	0.69^B^ ± 0.45	−0.36^A^ ± 0.15	0.99^A^ ± 0.64
Day 4	L*	53.32^B^ ± 0.39	55.20^B^ ± 0.48	56.22^A^ ± 0.83	47.30^C^ ± 0.80	56.91^B^ ± 0.60	58.47^B^ ± 0.76	57.72^BC^ ± 1.09	58.74^AB^ ± 0.55	56.79^B^ ± 0.60	58.88^AB^ ± 0.51
	a*	10.61^C^ ± 0.21	10.75^C^ ± 0.35	10.34^C^ ± 0.19	13.47^C^ ± 0.27	10.15^C^ ± 0.27	−3.30^D^ ± 0.06	−3.53^D^ ± 0.16	−3.90^C^ ± 0.12	−4.11^D^ ± 0.15	−3.89^C^ ± 0.13
	b*	9.22^B^ ± 0.19	9.35^B^ ± 0.11	9.87^AB^ ± 0.25	6.76^C^ ± 0.07	9.87^C^ ± 0.09	2.19^CD^ ± 0.11	−0.57^BC^ ± 0.09	0.29^C^ ± 0.17	−1.74^BC^ ± 0.16	−0.36^B^ ± 0.43
Day 5	L*	52.86^B^ ± 1.24	53.91^C^ ± 0.84	54.01^B^ ± 0.83	46.87^C^ ± 0.76	56.34^B^ ± 1.15	58.09^B^ ± 0.17	56.77^C^ ± 1.12	56.95^C^ ± 1.12	56.44^B^ ± 0.42	57.59^B^ ± 1.61
	a*	10.35^C^ ± 0.39	10.26^C^ ± 0.24	9.45^D^ ± 0.18	13.49^C^ ± 0.36	9.97^C^ ± 0.50	−3.27^D^ ± 0.10	−3.40^CD^ ± 0.07	−3.78^C^ ± 0.05	−3.99^D^ ± 0.10	−3.74^C^ ± 0.09
	b*	9.03^B^ ± 0.30	9.19^B^ ± 0.23	9.68^BC^ ± 0.35	6.69^C^ ± 0.02	9.70^C^ ± 0.13	1.96^D^ ± 0.02	−0.78^C^ ± 0.15	0.30^C^ ± 0.07	−1.86^C^ ± 0.25	−0.56^B^ ± 0.07

*Note*: Values are expressed as mean ± standard deviation. For each color characteristic, the mean followed by different letters differs significantly (*p* < 0.05) for each sample on different storage days.

Abbreviations: DC_B, dextrose‐sweetened cheesecake filling – blue; DC_P, dextrose‐sweetened cheesecake filling – pink; FC_B, fructose‐sweetened cheesecake filling – blue; FC_P, fructose‐sweetened cheesecake filling – pink; SoC_B, sorbitol‐sweetened cheesecake filling – blue; SoC_P, sorbitol‐sweetened cheesecake filling – pink; SuC_B, sucrose‐sweetened cheesecake filling – blue; SuC_P, sucrose‐sweetened cheesecake filling – pink; XC_B, xylitol‐sweetened cheesecake filling – blue; XC_P, xylitol‐sweetened cheesecake filling – pink.

Regarding the L* value, which indicates the lightness (0 indicates black and 100 indicates white), for both cheesecake fillings, the value decreased on Day 2, increased slightly on Day 3, and gradually decreased on Days 4 and 5. Generally, insignificant values were observed between Days 1 and 3 of storage, except FC_B and DC_P and DC_B. Similarly, there were no significant variations between Days 4 and 5 of storage, except FC_P, SoC_P, and SoC_B. The highest lightness value was recorded on Day 3 for the blue filling sweetened with fructose, −61.93, and the lowest for the pink filling with dextrose, 46.87. For the values of the parameter a*, all the red fillings recorded positive values, which indicates a shade of red. Similar to the results of the L*, the same trend of decreasing (Day 2), increasing (Day 3), and decreasing again (Days 4 and 5), and insignificant values between Days 4 and 5, except SoC_P, were observed. On the other hand, the values of the parameter a* were negative for the blue filling, indicating a green shade. They varied between −0.54 and −4.11 for DC_B on Day 3, respectively, 4 of storage.

The b* values were generally positive and higher for the pink fillings, reflecting a predominance of yellow hues, whereas the blue fillings exhibited lower b* values, indicative of a tendency toward blue shades. Only FC_B, DC_B, and XC_B demonstrated negative b* values, suggesting that the presence of sweeteners influences the development of samples with bluer coloration. For all samples, regardless of the day of storage, pink filling led to a decrease in the color parameters of the samples due to the darker color compared to the use of phycocyanin. Similar values were obtained in the study carried out by [50] on the ice cream formulated with *Spirulina platensis* for the L* values, but on the other hand, they obtained higher values for a* and b*, probably due to the different ingredients and production technology.

In the case of the section color of the crust, the highest value of the L* was registered for the crust formulated with xylitol on Day 1, 41.59, and the lowest for the crust with dextrose on Day 5, 30.49 (Table 3). Using different sweeteners influenced the values of parameters L* and b*, thus, on storage Days 1, 2, and 5, the values of the parameters decreased as follows: XC > SuC > SoC > FC > DC. For the external surface of the cheesecake crust, a similar decreasing tendency on Days 2 and 3, increasing on Day 4, followed by another decrease on Day 5 for the L* value observed for samples sweetened with sorbitol and xylitol; for the a*, the crusts obtained with fructose, sorbitol, and dextrose showed a decreasing trend on Day 2, increase on Day 3, followed by a decrease on Days 4 and 5. The only sample that did not show statistically significant differences in the color attributes during the 5 days of the study was the crust obtained with dextrose. Conversely, the crust made with xylitol showed no significant changes between Day 2 and Day 5. In contrast, samples containing fructose and sorbitol were only observed on Days 4 and 5. The section color varied more than the external surface color.

**TABLE 3:** Color characteristics for the section and external surface of the cheesecake crust.

		Section					External surface				
		SuC_C	FC_C	SoC_C	DC_C	XC_C	SuC_C	FC_C	SoC_C	DC_C	XC_C
Day 1	L*	38.23^AB^ ± 0.35	34.79^A^ ± 1.26	36.46^A^ ± 1.59	33.60^B^ ± 0.89	41.59^A^ ± 0.62	32.34^D^ ± 0.47	37.87^A^ ± 1.59	39.43^A^ ± 1.81	35.50^A^ ± 0.74	40.38^A^ ± 0.80
	a*	16.92^AB^ ± 0.71	9.21^B^ ± 1.52	8.98^C^ ± 1.83	10.74^A^ ± 2.68	19.83^A^ ± 2.62	16.63^B^ ± 1.77	19.14^B^ ± 3.48	17.28^A^ ± 3.22	10.94^A^ ± 1.75	15.47^A^ ± 3.22
	b*	24.45^AB^ ± 0.65	16.30^A^ ± 2.12	18.85^A^ ± 1.72	15.28^B^ ± 1.64	33.10^A^ ± 1.71	15.18^C^ ± 1.28	23.78^A^ ± 2.72	27.56^A^ ± 3.59	17.85^A^ ± 1.82	31.34^A^ ± 2.76
Day 2	L*	37.38^BC^ ± 0.95	33.55^AB^ ± 0.88	34.82^BC^ ± 0.48	32.21^BC^ ± 0.67	37.53^BC^ ± 0.60	40.53^A^ ± 0.56	36.04^AB^ ± 0.59	38.84^AB^ ± 1.56	34.67^A^ ± 0.76	38.62^B^ ± 1.07
	a*	14.54^B^ ± 2.90	8.35^B^ ± 0.68	12.31^B^ ± 0.67	12.14^A^ ± 1.49	7.80^C^ ± 1.33	21.17^A^ ± 0.52	15.81^B^ ± 2.61	16.70^A^ ± 2.07	9.08^A^ ± 1.47	16.99^A^ ± 1.44
	b*	22.13^BC^ ± 2.42	15.68^AB^ ± 1.48	17.38^AB^ ± 0.87	14.24^B^ ± 1.02	22.40^CD^ ± 0.78	28.42^A^ ± 0.74	20.36^B^ ± 1.24	26.61^AB^ ± 2.93	15.88^A^ ± 0.83	25.79^B^ ± 2.59
Day 3	L*	38.75^A^ ± 0.43	31.57^C^ ± 0.63	35.33^AB^ ± 0.46	31.97^CD^ ± 1.02	37.44^C^ ± 1.22	34.92^C^ ± 1.58	36.94^AB^ ± 1.83	37.00^AB^ ± 2.16	34.81^A^ ± 1.26	38.28^B^ ± 1.07
	a*	18.08^A^ ± 1.19	15.14^A^ ± 2.45	16.39^A^ ± 1.56	10.48^A^ ± 3.35	13.47^B^ ± 2.06	19.49^AB^ ± 3.31	23.06^A^ ± 1.10	17.20^A^ ± 1.86	10.90^A^ ± 2.33	15.30^A^ ± 1.76
	b*	25.54^A^ ± 1.02	14.49^AB^ ± 1.17	18.85^A^ ± 0.77	13.65^B^ ± 1.54	23.86^BC^ ± 2.87	19.60^B^ ± 3.13	21.85^AB^ ± 2.21	24.20^AB^ ± 3.96	16.81^A^ ± 2.55	25.88^B^ ± 3.38
Day 4	L*	36.88^C^ ± 0.76	32.51^BC^ ± 1.09	34.17^BC^ ± 0.92	35.10^A^ ± 0.99	35.79^D^ ± 0.68	38.60^B^ ± 1.02	35.46^B^ ± 0.98	37.95^AB^ ± 0.77	35.85^A^ ± 1.09	38.50^B^ ± 0.77
	a*	10.34^C^ ± 1.69	9.06^B^ ± 1.86	10.86^BC^ ± 0.96	10.18^A^ ± 1.36	9.33^C^ ± 1.12	19.20^AB^ ± 0.88	18.66^B^ ± 1.13	14.77^A^ ± 1.80	11.23^A^ ± 2.45	16.14^A^ ± 0.85
	b*	20.64^C^ ± 2.26	14.82^AB^ ± 2.56	16.29^B^ ± 0.99	18.11^A^ ± 2.29	20.48^D^ ± 1.48	25.63^A^ ± 2.18	19.94^B^ ± 1.24	25.35^AB^ ± 2.68	18.52^A^ ± 1.64	27.20^AB^ ± 2.31
Day 5	L*	37.73^ABC^ ± 0.87	31.36^C^ ± 0.31	33.40^C^ ± 0.89	30.49^D^ ± 0.73	38.75^B^ ± 0.48	37.09^B^ ± 0.52	35.20^B^ ± 0.86	36.22^B^ ± 1.57	35.66^A^ ± 0.99	38.45^B^ ± 0.93
	a*	17.14^AB^ ± 1.46	10.21^B^ ± 1.36	12.40^B^ ± 0.99	8.45^A^ ± 1.29	14.29^B^ ± 1.31	17.25^B^ ± 1.16	17.67^B^ ± 1.75	13.28^A^ ± 2.62	10.16^A^ ± 2.36	15.11^A^ ± 1.96
	b*	23.55^ABC^ ± 1.71	13.09^B^ ± 0.77	15.67^B^ ± 0.69	10.83^C^ ± 0.55	25.51^B^ ± 1.59	22.39^B^ ± 1.05	19.11^B^ ± 1.15	21.28^B^ ± 3.47	16.87^A^ ± 2.38	27.12^AB^ ± 2.29

*Note*: Values are expressed as mean ± standard deviation. For each color characteristic, mean followed by different letters differs significantly (*p* < 0.05) for each sample on different storage days.

Abbreviations: DC_C, dextrose‐sweetened cheesecake cake; FC_C, fructose‐sweetened cheesecake cake; SoC_C, sorbitol‐sweetened cheesecake cake; SuC_C, sucrose‐sweetened cheesecake cake; XC_C, xylitol‐sweetened cheesecake cake.

These findings align with previous studies evaluating betalain stability in thermally processed and refrigerated food systems. For example, Chandran et al. (2014) reported that betacyanin solutions subjected to mild thermal treatments (60–70°C) exhibited significant reductions in chroma (C*) and a* values, with corresponding increases in L*, closely matching the trends observed in our baked cheesecake matrices [51]. Similarly, studies by Czyzowska et al. (2020) demonstrated that while betalains are relatively stable under cold storage, initial thermal exposure results in notable color losses, particularly in systems lacking strong water‐binding matrices [52]. Notably, the retention of betacyanin color in our xylitol‐ and sorbitol‐sweetened cheesecakes was superior to that reported by Rocha et al. (2022) for betalain‐enriched yogurt beverages, where higher water activity promoted greater color degradation over 14 days at 4°C. This difference may be attributed to the lower water mobility and oxygen diffusion in the cheesecake matrix, enhanced by polyol‐rich sweetener systems [53].

### 3.5 Antioxidant Activity of Cheesecakes

The antioxidant activity was evaluated separately for the cheesecake filling and the cake base. Individual extracts were prepared from each component using the same extraction protocol, and the DPPH and ABTS assays were applied on Days 1, 3, and 5. Both assays underscore the ability of these compounds to mitigate oxidative stress through electron or hydrogen atom transfer mechanisms. Comparing the results from both assays, the study aims to provide a comprehensive view of the antioxidant capacities of cheesecake components.

**FIGURE 3:**
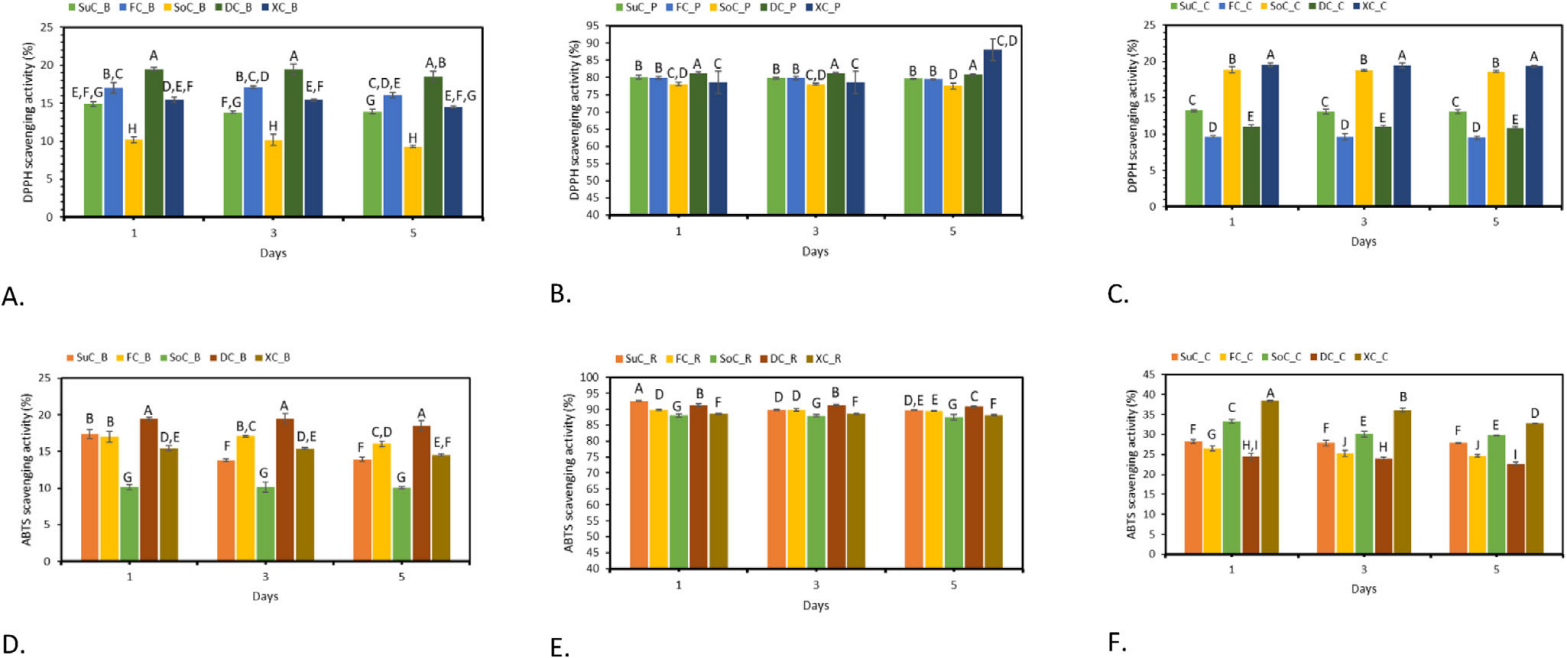
Antioxidant activity evaluation with DPPH (A–C) and ABTS (D–F) assays for cheesecake fillings and cake. Values are expressed as mean ± standard deviation. Mean followed by different letters differs significantly (*p* < 0.05). ABTS, 2,2′‐azinobis‐(3‐ethylbenzothiazoline‐6‐sulfonic acid); DC_B, dextrose‐sweetened cheesecake filling – blue; DC_C, dextrose‐sweetened cheesecake cake; DC_P, dextrose‐sweetened cheesecake filling – pink; DPPH, 1,1‐diphenyl‐2‐picrylhydrazyl; FC_B, fructose‐sweetened cheesecake filling – blue; FC_C, fructose‐sweetened cheesecake cake; FC_P, fructose‐sweetened cheesecake filling – pink; SoC_B, sorbitol‐sweetened cheesecake filling – blue; SoC_C, sorbitol‐sweetened cheesecake cake; SoC_P, sorbitol‐sweetened cheesecake filling – pink; SuC_B, sucrose‐sweetened cheesecake filling – blue; SuC_C, sucrose‐sweetened cheesecake cake; SuC_P, sucrose‐sweetened cheesecake filling – pink; XC_B, xylitol‐sweetened cheesecake filling – blue; XC_C, xylitol‐sweetened cheesecake cake; XC_P, xylitol‐sweetened cheesecake filling – pink.

For the pink filling (Figure 3A, D), which contains anthocyanins, both assays showed significant variation in antioxidant activity depending on the sweetener used. The DC_P exhibited the highest antioxidant activity in the DPPH and ABTS assays. This indicates that dextrose significantly enhances the antioxidant capacity of anthocyanins. The FC_P demonstrated slightly lower antioxidant activity than dextrose but higher than the other sweeteners. During the cheesecake shelf life, each sweetener demonstrated consistent maintenance of antioxidant activity, with no significant decreases observed (*p* > 0.05). These results suggest that dextrose and fructose are more effective in enhancing the antioxidant capacity of the pink cheesecake filling compared to xylitol and sorbitol, results sustained by the literature [54]. The research also indicates that the quantity of sugar influences the free radical scavenging capacity, with rutinose‐containing anthocyanins exhibiting higher antioxidant capacity than glucose‐containing anthocyanins [54]. Additionally, glycosylation of anthocyanins has been found to impact their radical scavenger activity, with aglycone forms showing higher activity than glycosylated forms due to the reduction in the ability of anthocyanin radicals to delocalize electrons [54, 55].

The blue filling (Figure 3B, E), containing phycocyanin, also showed consistent antioxidant activity variations with different sweeteners in both assays. The DC_B demonstrated the highest antioxidant activity, indicating a strong enhancement effect by dextrose. Similar to the pink filling, the XC_B and the SoC_B exhibited the lowest antioxidant activities in both assays. These findings indicate that dextrose and fructose significantly enhance the antioxidant properties of the phycocyanin cheesecake filling more effectively than xylitol and sorbitol.

Dextrose and fructose consistently showed the highest enhancement of antioxidant activities across both cheesecake fillings, likely due to their interactions with antioxidant compounds such as phycocyanin and anthocyanins. Dextrose, a simple sugar, and fructose, a natural fruit sugar, are more effective in enhancing antioxidant activity [56, 57]. The enhanced antioxidant activity can be mechanistically attributed to the high reducing potential of these monosaccharides, which possess free carbonyl groups. These reactive groups facilitate electron or hydrogen atom donation, directly scavenging free radicals (DPPH• and ABTS+•) through single‐electron transfer and hydrogen atom transfer mechanisms [58, 59, 60]. Furthermore, reducing sugars can interact with anthocyanins and phycocyanins via weak noncovalent interactions (van der Waals forces), stabilizing the pigment structure and preventing oxidative degradation [60, 61]. For anthocyanins, glycosylation already improves their radical scavenging properties by enhancing solubility; additional interactions with reducing sugars may further stabilize the flavylium cation form, the most antioxidant‐active species under acidic conditions [59, 62]. Reducing sugars also participate in the early stages of the Maillard reaction, producing Maillard reaction products [63]. Specifically, Amadori rearrangement products and low MW melanoidins generated under mild thermal conditions have potent free radical‐scavenging activity [64]. These findings have important implications for confectionery health benefits and functional features. Choosing sweeteners like dextrose or fructose can significantly boost the antioxidant properties of desserts, potentially offering health advantages such as reduced oxidative stress. However, further research is needed to elucidate the exact interaction mechanisms between these sweeteners and antioxidant compounds. Additionally, sensory evaluations are essential to ensure that the enhanced antioxidant capacity does not negatively impact the taste and texture.

For the cheesecake cake (Figure 3C, F) containing betacyanin, the trends observed were consistent in both the DPPH and ABTS assays. The XC_C showed the highest antioxidant activity (38.41%), followed by the SoC_C (33.28%). The results align with the phytochemical and color findings, highlighting sugar alcohols’ good protection against thermal degradation. The results also align with the literature demonstrating antioxidant activity in various systems. For example, Al‐Shahrani et al. (2013) findings indicate that xylitol had the highest antioxidant activity (0.3 Mesfer unit) with closer values to vitamin C (1.000 Mesfer unit) [65].

### 3.6 Rheological Measurements

**FIGURE 4:**
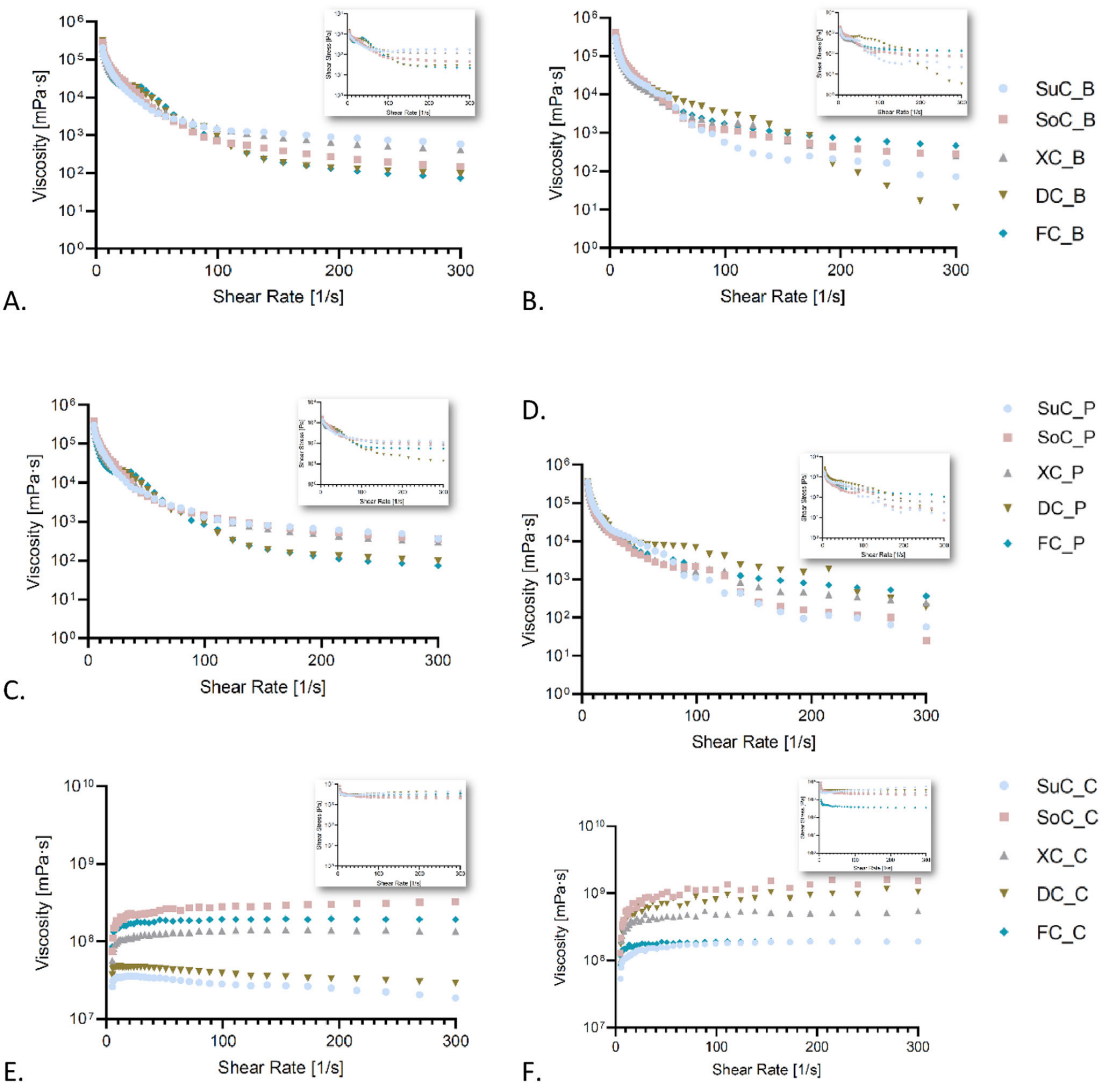
The viscosity of each filling of cheesecake Day 1 (A, C, and E) and 5 days of storage (B, D, and F). GraphPad Prism Version 8.0.1 (Graph Pad Software, Inc., San Diego, CA, USA). DC_B, dextrose‐sweetened cheesecake filling – blue; DC_C, dextrose‐sweetened cheesecake cake; DC_P, dextrose‐sweetened cheesecake filling – pink; FC_B, fructose‐sweetened cheesecake filling – blue; FC_C, fructose‐sweetened cheesecake cake; FC_P, fructose‐sweetened cheesecake filling – pink; SoC_B, sorbitol‐sweetened cheesecake filling – blue; SoC_C, sorbitol‐sweetened cheesecake cake; SoC_P, sorbitol‐sweetened cheesecake filling – pink; SuC_B, sucrose‐sweetened cheesecake filling – blue; SuC_C, sucrose‐sweetened cheesecake cake; SuC_P, sucrose‐sweetened cheesecake filling – pink; XC_B, xylitol‐sweetened cheesecake filling – blue; XC_C, xylitol‐sweetened cheesecake cake; XC_P, xylitol‐sweetened cheesecake filling – pink.

In the present study, the viscosity of each cheesecake level was evaluated at shear rates ranging from 5 to 300 1/s at a storage temperature of 4°C (Figure 4). The viscosity of cheesecake fillings was assessed to determine their physical stability and texture, which are vital for consumer preference [66]. Viscosity indicates that sweeteners influence the structure of the matrix and the protection of pigments, as increased viscosity can minimize pigment degradation by restricting molecular movement and oxidative reactions [66, 67]. The rheological analysis also shows both the quality of texture and the retention of natural colorings in storage.

The perception of thickness during oral consumption is closely linked to this measurement, making it an effective indicator of functionality. Both cheesecake fillings exhibited shear‐thinning (pseudoplastic) behavior, consistently decreasing viscosity with increasing shear rates, resulting in lower resistance. The rapid reduction in the samples’ viscosity indicates that, initially, the small particles form a tangled mass when the sample is at rest. As the shear rate increases, these particles align, decreasing viscosity. This behavior aligns with findings from similar studies on various food products, confirming that particle alignment under shear stress is a common phenomenon influencing viscosity [68, 69]. The lower part of the cake presented dilatant (shear‐thickening) behavior. These dilatant samples exhibited increased viscosity with rising shear rates, but the viscosity remained generally constant at shear rates above 30 1/s. Shear thickening occurs when hydrodynamic shear forces overcome Brownian forces, forming transient aggregates called hydroclusters, which thicken the sample [69].

Viscosity and shear stress appeared higher before storage in both fillings sweetened with sucrose, but afterward, they decreased considerably (*p* < 0.05). Fructose exhibited a different behavior; its viscosity was low before storage but increased significantly afterward. Therefore, sweeteners dictate product texture, viscosity, and freezing point. Their impact extends beyond mere sweetness, as they significantly alter the mouthfeel and consistency, which are critical factors in determining consumer satisfaction. By modifying a product's physical properties, sweeteners can enhance or detract from its overall appeal, thereby making them one of the most influential ingredients in consumer acceptance [70]. The balance and choice of sweeteners are thus essential in product formulation to meet consumer expectations and preferences.

### 3.7 Sensory Analysis

**FIGURE 5:**
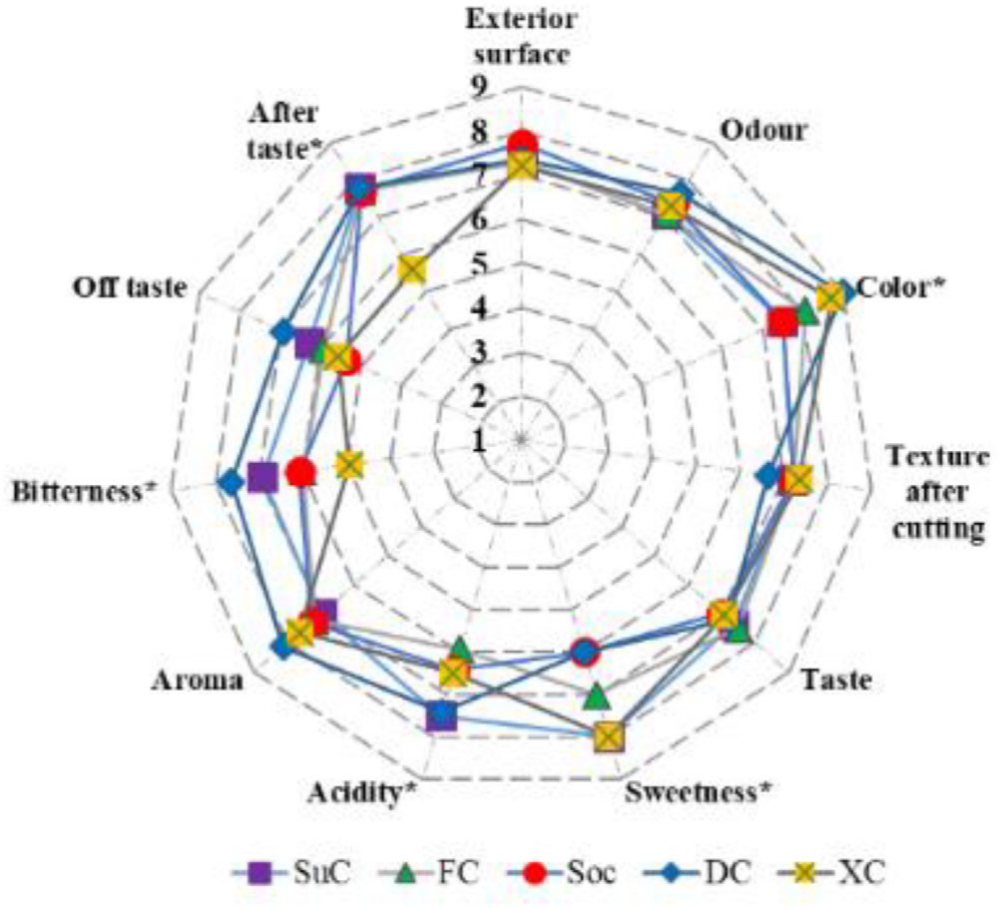
Sensory evaluation of the cheesecakes. Means ± standard deviation followed by * indicates statistically significant differences between the types of cheesecakes tested according to Tukey's test (*p* < 0.05). DC, dextrose cheesecake; FC, fructose‐sweetened cheesecake; SoC, sorbitol cheesecake; SuC, sucrose sweetened cheesecake; XC, xylitol cheesecake.

This research included volunteer customers who expressed interest in evaluating innovative food products manufactured using natural components. When developing a new food product, conducting sensory testing with consumers is crucial to assess its acceptability before launching it on the market. The samples were stored at ±4°C, and the sensory evaluation was performed on the day after their production. Figure 5 presents the radar chart (expressed as the mean of the hedonic scores) for all the sensory attributes of cheesecakes formulated with different sweeteners. All samples had positive hedonic ratings over 5, indicating that they may be marketed, except XC, due to its bitterness. Among all attributes, the evaluators noticed significant differences between samples only in color, sweetness, acidity, bitterness, and aftertaste. The highest acceptance for color was recorded for DC (9 ± 0.70). The use of different sweeteners significantly influences the perception of sweet taste, with scores of 6 for SoC and DC, 7 for FC, and 8 for SuC and XC. The aftertaste was generally pleasant, with an average hedonic score of around 7.70, except for the XC –5.57. The acidity was significantly perceived in the FC, SoC, and SC samples, while bitterness was especially noted in the XC sample. Similar hedonic scores were obtained for cheesecakes with sucrose and fructose [71].

## 4 Conclusion

In summary, the study results revealed that phycocyanin, anthocyanins, and betacyanin affect cheesecake phytochemical and physical stability differently. Phycocyanin proved the most stable, effectively preserving its vibrant blue color under refrigerated conditions. Despite initially presenting strong coloration, anthocyanins exhibited significant degradation over time, even though the pH of the cheesecakes remained stable throughout the monitored periods. Betacyanin underwent color degradation due to temperature exposure but displayed good stability during cheesecake shelf life. Additionally, the choice of sweetener in cheesecake formulations significantly influenced various nutritional, sensory, and stability attributes. Dextrose and fructose were identified as the most effective stabilizers for phycocyanin and anthocyanins, whereas xylitol demonstrated superior efficacy in preserving the stability of betacyanin. This observation can be attributed to these pigments’ sensitivity to pH and temperature variations. On the other hand, future investigations are required regarding the biochemical and molecular pathways involved in the stability and activity of these pigments and sweeteners interactions. Also, evaluating a broader spectrum of sweeteners, such as stevia, erythritol, and monk fruit extract, should be included to provide a more comprehensive understanding of their effects on phytochemical stability and antioxidant activity.

## Conflicts of Interest

The authors declare no conflicts of interest.

## Supporting information




**Supporting Information file 1**: mnfr70191‐sup‐0001‐SuppMat.docx

## Data Availability

The data that support the findings of this study are available from the corresponding author upon reasonable request.
