# Supplementary material for: Exploring the Phytochemical and Physical Stability of Phycocyanin, Anthocyanins, and Betacyanin in a Cheesecake Product
Source: Mol Nutr Food Res. 2025 Aug 10;69(21):e70191. doi: 10.1002/mnfr.70191 (PMC12581751; doi:10.1002/mnfr.70191)
Supplement: Supplementary file 1 — Supporting Information file 1: mnfr70191‐sup‐0001‐SuppMat.docx [file MNFR-69-e70191-s001.docx]

Exploring the phytochemical and physical stability of phycocyanin, anthocyanins, and betacyanin in a cheesecake product

*Cristina Selin^1^†, Anda Tanislav^1,3^, Laura Stan^1^, Bernadette-Emőke Teleky^1^, Călina Ciont (Nagy)^1,2^†, Florica Ranga^1^, Vlad Mureşan^3^, Andruţa Mureşan^3^, Dan Cristian Vodnar^1^, Mihaela Mihai^1^, Ionela Daniela Morariu^4^ and Oana Lelia Pop^1,2^**

^1^ Department of Food Science, University of Agricultural Sciences and Veterinary Medicine, 400372, Cluj-Napoca, Romania;

^2^ Molecular Nutrition and Proteomics Laboratory, Institute of Life Sciences, University of Agricultural Sciences and Veterinary Medicine, 400372, Cluj-Napoca, Romania;

^3^ Department Food Technology, University of Agricultural Sciences and Veterinary Medicine, 400372, Cluj-Napoca, Romania;

^4^ Department of Environmental and Food Chemistry, University of Medicine and Pharmacy "Grigore T. Popa", 700115, Iasi, Romania;

*Correspondence: [oana.pop@usamvcluj.ro](mailto:oana.pop@usamvcluj.ro) (O.L.P.);

† - These authors contributed equally to this work

Table 1. Composition of cheesecakes with varied sweetener additions

| **Ingredients** | **SC** | **FC** | **SoC** | **DC** | **XC** |
| --- | --- | --- | --- | --- | --- |
| **(g)** |  |  |  |  |  |
| Sucrose | 15 | - | - | - | - |
| Fructose | - | 15 | - | - | - |
| Sorbitol | - | - | 15 | - | - |
| Dextrose | - | - | - | 15 | - |
| Xylitol | - | - | - | - | 15 |
| Whipping cream 38% fat | 23 | | | | |
| Quark cheese | 13 | | | | |
| Yogurt 2% fat | 7 | | | | |
| Wheat flour | 15 | | | | |
| Beetroots *(Beta vulgaris* L*.)*  pulp | 10 | | | | |
| Sunflower oil | 10 | | | | |
| Hydration water for gelatin | 1.5 | | | | |
| Egg albumen | 2 | | | | |
| Haskap berries (*Lonicera caerulea* L. var.Vostorg), freeze-dried | 0.4 | | | | |
| Beef gelatin | 1 | | | | |
| Egg yolk | 1 | | | | |
| Phycocyanin powder | 0.4 | | | | |
| Baking powder | 0.3 | | | | |
| Alcoholic vanilla extract | 0.4 | | | | |

SuC- sucrose sweetened cheesecake; FC- fructose sweetened cheesecake; SoC- sorbitol cheesecake; DC-dextrose cheesecake; XC-xylitol cheesecake

Figure S1. Chromatograms of anthocyanins from the pink filing of the cheesecake colored with freeze dried haskap berry (*Lonicera caerulea* L.)

MS spectrum of peak Rt = 11.02 min; [M+H] = 449, Cyanidin-glucoside

Table 2. Microbial development on the cheesecake samples (log CFU/g)

| **Cheesecake Samples** | **Storage period (days)** | **Bacterial strains** | | | | |
| --- | --- | --- | --- | --- | --- | --- |
|  |  | **Y&M** | ***Enterobacteriaceae*** | ***E.coli*** | ***S. aureus*** | **Coliform bacteria** |
| **SuC** | 2 | n.b | n.b | n.b | n.b | n.b |
|  | 5 | n.b | n.b | n.b | n.b | n.b |
|  | 7 | n.b | n.b | n.b | n.b | n.b |
| **FC** | 2 | n.b | n.b | n.b | n.b | n.b |
|  | 5 | 1.15^B^ ± 0.21 | n.b | n.b | n.b | n.b |
|  | 7 | 1.24^B^ ± 0.34 | n.b | n.b | n.b | n.b |
| **SoC** | 2 | n.b | n.b | n.b | n.b | n.b |
|  | 5 | n.b | n.b | n.b | n.b | n.b |
|  | 7 | 1.15^B^ ± 0.21 | n.b | n.b | n.b | n.b |
| **DC** | 2 | n.b | n.b | n.b | n.b | n.b |
|  | 5 | n.b | n.b | n.b | n.b | n.b |
|  | 7 | n.b | n.b | n.b | n.b | n.b |
| **XC** | 2 | n.b | n.b | n.b | n.b | n.b |
|  | 5 | n.b | n.b | n.b | n.b | n.b |
|  | 7 | 1.39^B^ ± 0.12 | n.b | n.b | n.b | n.b |

All data are the mean ± SD of three independent determinations. Mean followed by different letters in the same column differs significantly (*p* < 0.05). SuC- sucrose sweetened cheesecake; FC- fructose-sweetened cheesecake; SoC- sorbitol sweetened cheesecake; DC-dextrose sweetened cheesecake; XC-xylitol sweetened cheesecake; n.b. – no bioactivity
